# Community norms of the Eating Pathology Symptoms Inventory (EPSI) in cisgender sexual minority adults

**DOI:** 10.1007/s40519-025-01742-3

**Published:** 2025-04-04

**Authors:** Jason M. Nagata, Christopher D. Otmar, Christopher M. Lee, Emilio J. Compte, Jason M. Lavender, Tiffany A. Brown, Kelsie T. Forbush, Annesa Flentje, Micah E. Lubensky, Juno Obedin-Maliver, Mitchell R. Lunn

**Affiliations:** 1 Department of Pediatrics, University of California, San Francisco, 550 16th Street, 4th Floor, Box 0503, San Francisco, CA 94143 USA; 2 Eating Behavior Research Center, School of Psychology, Universidad Adolfo Ibáñez, Santiago, Chile; 3 Research Department, Comenzar de Nuevo, Monterrey, México; 4 Military Cardiovascular Outcomes Research Program (Micor), Department of Medicine, Uniformed Services University of the Health Sciences, Bethesda, MD USA; 5 The Metis Foundation, San Antonio, TX USA; 6 Department of Psychological Sciences, Auburn University, Auburn, AL USA; 7 Department of Clinical Child Psychology, University of Kansas, Lawrence, KS USA; 8 The PRIDE Study/PRIDEnet, Stanford University School of Medicine, Stanford, CA USA; 9 Department of Community Health Systems, University of California, San Francisco, San Francisco, CA USA; 10 Department of Psychiatry and Behavioral Sciences, Alliance Health Project, University of California, San Francisco, San Francisco, CA USA; 11 Department of Obstetrics and Gynecology, Stanford University School of Medicine, Stanford, CA USA; 12 Department of Epidemiology and Population Health, Stanford University School of Medicine, Stanford, CA USA; 13 Division of Nephrology, Department of Medicine, Stanford University School of Medicine, Stanford, CA USA

**Keywords:** Eating disorders, Sexual minority, Community norms, LGBTQIA + health, LGBT, LGBTQ

## Abstract

**Purpose:**

Cisgender sexual minority adults are at elevated risk for eating disorders; however, community norms for certain eating disorder measures are lacking for this population. This study aimed to establish community norms for the Eating Pathology Symptom Inventory (EPSI) among cisgender gay, lesbian, bisexual + (individuals who identify as bisexual or pansexual; bi +) adults.

**Methods:**

Cross-sectional data were analyzed from 2062 cisgender participants—including 925 gay men, 573 lesbian women, 116 bi + men, and 448 bi + women—enrolled in The PRIDE Study, a national longitudinal cohort of sexual and gender minority adults in the United States. Participants completed the EPSI, and descriptive statistics were calculated for the eight EPSI scales.

**Results:**

We report mean scores, standard deviations, medians, interquartile ranges, and percentile ranks for the eight EPSI scales within cisgender sexual minority populations. Distinct patterns of eating-pathology symptoms were evidenced among the cisgender sexual minority groups. Cisgender bi + women exhibited the highest scores for Body Dissatisfaction, Binge Eating, and Restricting compared to other groups, whereas cisgender bi + men reported the highest scores for Excessive Exercise. Cisgender gay men had significantly higher scores for Muscle Building and Negative Attitudes Toward Obesity compared to other groups.

**Conclusion:**

These findings offer valuable benchmarks for interpreting EPSI scores in the assessment and treatment of eating disorders among cisgender sexual minority individuals.

*Level of evidence*: Level V: based on descriptive results.

## Introduction

Eating disorders are marked by disturbances in eating behaviors or maladaptive behaviors to control body weight/shape, which can lead to severe impairments in physical health and psychosocial functioning [1, 2]. In addition to physical signs (e.g., nutritional deficiencies), disordered-eating symptoms include cognitive (e.g., excessive concern or preoccupation with body weight or shape), behavioral (e.g., binge eating, purging, excessive exercise), and affective (e.g., intense fear of weight gain, guilt about binge eating) disturbances. These disorders often emerge during adolescence or early adulthood, affecting a diverse spectrum of individuals, although they are most frequently diagnosed in young adult heterosexual women [1, 3]. Given the serious mental and physical health impacts of eating disorders, including significantly elevated rates of morbidity and mortality [4], providers must have appropriate measures and relevant norms to accurately assess and contextualize the severity of disordered-eating symptoms, particularly in at-risk populations.

Eating disorders are frequently diagnosed in young adult women, with some studies indicating that they are more commonly reported among heterosexual women compared to their sexual minority peers [3]. However, cisgender sexual minority adults also experience higher rates of eating disorders and disordered eating behaviors than their heterosexual peers, likely due to a combination of stigma, societal bias, minority stress, and other social pressures [5, 6]. Although elevated eating-disorder risk is well-documented [7–9], few studies have examined eating-disorder pathology within specific cisgender sexual minority subgroups—such as gay, lesbian, bisexual, queer, and questioning adults. Within the sexual minority community, the prevalence of specific forms of disordered eating psychopathology may differ by sexual minority subgroup. For instance, research has indicated that (presumed cisgender) gay men and bisexual women report higher rates of anorexia nervosa and bulimia nervosa compared to presumed heterosexual individuals and other sexual and gender minority (SGM) subgroups [9–11]. Information stratified by subgroup can be particularly helpful in clinical settings, particularly when providers are utilizing validated measures to assess eating disorders. The ability to reference descriptive norms data can help clinicians interpret scores on specific measures and scales to inform treatment and discharge planning.

Recent work has sought to provide validation and community norms for certain eating disorder measures specifically in sexual minority populations. For example, the Eating Disorder Examination-Questionnaire has been validated in cisgender sexual minority adults, and normative data are available [12–14]. Preliminary findings also support the utility of a newer eating disorder measure, the Eating Pathology Symptom Inventory [15]. The EPSI is a 45-item, multidimensional self-report questionnaire designed to evaluate eating pathology symptoms across diverse populations, including weight and gender [15]. The measure comprises eight scales, including Body Dissatisfaction, Binge Eating, Cognitive Restraint, Purging, Restricting, Excessive Exercise, Muscle Building, and Negative Attitudes Toward Obesity [15]. Although the EPSI has been validated in a variety of populations, including gay and bisexual men [16], there are no published norms for the EPSI among cisgender sexual minority groups to our knowledge. Furthermore, no studies to date have explored EPSI community norms for cisgender lesbian and bisexual + (individuals who identify as bisexual or pansexual; bi +) women despite prior research highlighting higher rates of disordered-eating symptoms among women reporting bisexual attractions or uncertainty in terms of their attractions [17]. To address this gap in the literature, this investigation utilized data from a national sample of gender and sexual minority adults to provide community norms for the EPSI among cisgender gay men, lesbian women, and bi + adults. To characterize differences by cisgender sexual minority status, we conducted comparisons of median EPSI scores among gay men, lesbian women, bi + men, and bi + women.

## Method

This study utilized data from a subset of participants enrolled in The Population Research in Identity and Disparities for Equality (PRIDE) Study—a national, longitudinal cohort investigating the health and well-being of SGM adults across the United States. Eligibility criteria for inclusion required participants to have completed the ‘Eating and Body Image 2023’ survey, be at least 18 years old, reside in the U.S. or its territories, and be able to read and understand an English-language survey. Detailed information on The PRIDE Study’s community-engaged research design and recruitment methods have been reported elsewhere [18, 19]. Between July 2023 and January 2024, a total of 4729 participants from The PRIDE Study completed the ‘Eating and Body Image 2023’ ancillary survey. All participants were enrolled in The PRIDE Study, a well-established longitudinal cohort in which participants have completed annual surveys and ancillary studies for multiple years, reducing the likelihood of inattentive responding. To address concerns about duplicate responses, The PRIDE Study employs a robust participant authentication system that prevents multiple submissions from the same individual. Each survey invitation is linked to a unique, participant-specific survey URL that requires authentication through The PRIDE Study Portal. Participants must log into their personalized dashboard to access the survey, and once completed, it is removed from their dashboard, ensuring they cannot take it again. Additionally, each survey response is stored with a unique participant identifier (PID) within the Qualtrics system. Any responses without an associated PID are excluded from analysis, though no such cases have been observed to date. These safeguards effectively prevent duplicate responses without requiring additional manual validation steps.

### Inclusion criteria and study population

This study was specifically interested in the experiences of cisgender sexual minority adults. Therefore, inclusion criteria included identifying as a cisgender gay man, lesbian woman, bi + man, or bi + woman. Of the 4,729 participants who completed the survey, 2,062 (approximately 43.6%) met the inclusion criteria. The analytical sample comprised 925 cisgender gay men (44.9%), 573 cisgender lesbian women (27.8%), 116 cisgender bi + men (5.6%), and 448 cisgender bi + women (21.7%).

### Participant recruitment and informed consent

Participants were recruited through a variety of channels, including community engagement efforts via PRIDEnet (a national network focused on engaging SGM communities), online advertising through blog posts and newsletters, distribution of The PRIDE Study-branded materials, outreach at conferences and events, social media campaigns, and word-of-mouth referrals. Data were collected using a secure, cloud-based digital platform accessible from any smartphone, tablet, or computer. As an incentive for participation, individuals who completed the survey were entered into a raffle to win one of 50 $40 gift cards. The Institutional Review Boards of Stanford University School of Medicine, the University of California, San Francisco, and WIRB-Copernicus Group (WCG) approved this study, and oversight was provided by The PRIDE Study’s Research Advisory Committee and Participant Advisory Committee. All participants provided written informed consent prior to participation.

## Measures

### Sexual orientation and gender identity

Sexual orientation and gender identity were determined using two single self-report items: “If you had to choose only one of the following terms, which best describes your current sexual orientation?” and “If you had to choose only one of the following terms, which best describes your current gender identity?” Participants in the present study were included if they selected “gay/lesbian” or “bisexual/pansexual” for sexual orientation and “cisgender man” or “cisgender woman” for gender identity. Individuals who did not provide responses relevant to this analysis were excluded.

### Eating Pathology Symptoms Inventory (EPSI)

As a comprehensive tool for assessing eating disorder psychopathology, the EPSI includes 45 self-report items divided into eight scales: Body Dissatisfaction (7 items), Binge Eating (8 items), Cognitive Restraint (3 items), Purging (6 items), Restricting (6 items), Excessive Exercise (5 items), Muscle Building (5 items), and Negative Attitudes Toward Obesity (5 items) [15]. Participants rated their experiences over the past four weeks on a five-point Likert-type scale ranging from 0 (“Never”) to 4 (“Very often”), with higher scores indicating more severe eating-related symptoms.

### Data analysis

Results are presented as percentiles, means (standard deviation), medians (interquartile range [IQR]), and percentages. The assumption of normality was not fulfilled among continuous variables (Shapiro–Wilk: *p* < 0.001); therefore, the Kruskal–Wallis test was used to assess sexual orientation group differences (gay men, lesbian women, bi + men, bi + women) and post hoc Dunn’s tests were used to examine pairwise differences. We conducted a power analysis using a one-way ANOVA framework (Cohen’s f) as an approximation for the Kruskal–Wallis test. Based on our actual subgroup sizes, this study was well-powered to detect medium and large effects across the four groups. We acknowledge, however, that we may have been underpowered to detect small differences, especially in the smallest subgroup (i.e., cisgender bi + men), and our analysis relies on rank-based differences in medians rather than mean-based effect sizes. All post hoc comparisons were adjusted for multiple comparisons using the Bonferroni correction. Minimal missing data were observed across the subsamples. All analyses were conducted using R (version 4.4.2) [20]. Descriptive statistics for continuous variables (mean, standard deviation, and range) were calculated using the ‘dplyr’ package (version 1.1.4) [21]. Categorical variable summaries (counts and percentages) were generated using the ‘gtsummary’ package (version 2.0.3) [22]. Dunn’s post hoc tests were performed with the ‘dunn.test’ package (version 1.3.6) [23]. Additional summary tables were created using the ‘tableone’ package (version 0.13.2) [24].

## Results

### Sociodemographic characteristics

Table 1 presents participant sociodemographic characteristics. Across all four groups, participants were predominantly White and college-educated. The mean age for bi + men was 49.16 years (*SD* = 15.60), bi + women had a mean age of 36.49 years (*SD* = 11.14), gay men were 52.76 years old on average (*SD* = 15.16), and lesbian women had a mean age of 48.21 years (*SD* = 17.04). Most participants identified as White, ranging from 87.93% to 94.76% across groups, with the remainder identifying with a race/ethnicity other than White (5.24% to 12.07%). Participants were allowed to select more than one race/ethnicity. A proportion of participants in each group identified as multiracial: 7.8% of bi + men, 10.5% of bi + women, 9.1% of gay men, and 7.9% of lesbian women reported multiple ethno-racial identities. Additionally, the majority of participants had completed at least a college degree with 82.8% to 90.8% having attained a college or higher education.

**Table 1:** Sociodemographic characteristics

	Cisgender gay men	Cisgender lesbian women	Cisgender bi + men	Cisgender bi + women	*p*
*N*	925	573	116	448	
Sociodemographic characteristics					
Age					
Mean (*SD*)	49.16 (15.60)	36.49 (11.14)	52.76 (15.16)	48.21 (17.04)	< 0.001
Race					0.005
American Indian/Alaska Native	1 (0.86%)	13 (2.90%)	27 (2.92%)	18 (3.14%)	
Asian	5 (4.31%)	23 (5.13%)	37 (4.00%)	13 (2.27%)	
Black/African American	10 (8.62%)	21 (4.69%)	40 (4.32%)	20 (3.49%)	
Hispanic/Latino	7 (6.03%)	27 (6.03%)	68 (7.35%)	20 (3.49%)	
Middle Eastern/North African	1 (0.86%)	3 (0.67%)	11 (1.19%)	3 (0.52%)	
Native Hawaiian/Pacific Islander	1 (0.86%)	0 (0.00%)	5 (0.54%)	2 (0.35%)	
White	102 (87.93%)	416 (92.86%)	826 (89.30%)	543 (94.76%)	
Other/unknown	2 (1.72%)	2 (0.45%)	9 (0.97%)	16 (2.79%)	
Education					
No schooling	0 (0.0%)	0 (0.0%)	0 (0.0%)	0 (0.0%)	< 0.001
Nursery to high school, no diploma	1 (0.9%)	0 (0.0%)	1 (0.1%)	5 (0.9%)	
High school graduate or equivalent	3 (2.6%)	16 (3.6%)	37 (4.0%)	13 (2.3%)	
Trade/technical/vocational training	2 (1.7%)	9 (2.0%)	14 (1.5%)	7 (1.2%)	
Some college	16 (13.8%)	54 (12.1%)	104 (11.2%)	54 (9.4%)	
2-year college degree	4 (3.4%)	28 (6.2%)	47 (5.1%)	23 (4.0%)	
4-year college degree	36 (31.0%)	152 (33.9%)	290 (31.4%)	149 (26.0%)	
Master’s degree	32 (27.6%)	121 (27.0%)	255 (27.6%)	198 (34.6%)	
Doctoral degree	11 (9.5%)	40 (8.9%)	77 (8.3%)	74 (12.9%)	
Professional degree	11 (9.5%)	28 (6.2%)	100 (10.8%)	49 (8.6%)	
Income					< 0.001
$0–$30,000	26 (23.2%)	156 (34.8%)	200 (22.0%)	148 (26.3%)	
$30,001–$60,000	31 (27.7%)	137 (30.6%)	219 (24.3%)	161 (28.4%)	
$60,001–$100,000	25 (22.3%)	97 (21.6%)	209 (23.2%)	139 (24.5%)	
$100,001–$150,000	14 (12.5%)	37 (8.3%)	190 (21.0%)	79 (13.9%)	
$150,001 +	18 (16.1%)	24 (5.3%)	160 (17.8%)	44 (7.9%)	

*p*-values for continuous variables were calculated using one-way ANOVA, and p-values for categorical variables were derived from Chi-square tests of independence. Participants were allowed to select more than one race/ethnicity, resulting in percentages that exceed 100%

### Eating Pathology Symptoms Inventory (EPSI)

Table 2 provides descriptive statistics (means, standard deviations, medians, interquartile ranges, and percentile ranks) for each EPSI scale. As shown in Table 3, several notable between-group differences emerged. Cisgender men (gay and bi +) reported lower Body Dissatisfaction compared to cisgender women (lesbian and bi +). Cisgender bi + men reported lower Binge Eating scores than the other groups, whereas cisgender bi + women showed lower Cognitive Restraint scores relative to all others. Although Purging scores did not differ significantly by group, cisgender bi + women reported slightly higher Restricting scores. Cisgender bi + men demonstrated the highest Excessive Exercise scores, followed by cisgender gay men, cisgender lesbian women, and cisgender bi + women. Negative Attitudes toward Obesity scores were most pronounced among cisgender gay men and bi + men. Muscle Building scores were elevated among cisgender gay men compared to the other subgroups. Complete Chi-square statistics and significance levels for these comparisons are provided in Table 3.

**Table 2:** Distribution of means, standard deviations, medians, interquartile ranges, and percentile ranks for the Eating Pathology Symptoms Inventory (EPSI) scales

Cisgender gay men (*N* = 925)								
	Body dissatisfaction	Binge eating	Cognitive restraint	Purging	Restricting	Excessive exercise	Negative attitudes toward obesity	Muscle building
*M* (*SD*)	11.11 (6.42)	8.32 (6.43)	5.08 (2.83)	0.84 (2.35)	4.42 (4.42)	4.82 (4.66)	6.64 (4.85)	4.37 (3.99)
Range	0–28	0–32	0–12	0–23	0–24	0–20	0–20	0–20
Rank								
5	2.00	0.00	0.00	0.00	0.00	0.00	0.00	0.00
10	3.00	1.00	1.00	0.00	0.00	0.00	0.00	1.00
15	4.00	2.00	2.00	0.00	0.00	0.00	1.00	1.00
20	5.00	2.00	2.00	0.00	1.00	0.00	2.00	1.00
25	6.00	3.00	3.00	0.00	1.00	1.00	3.00	2.00
30	7.00	4.00	3.20	0.00	1.00	1.00	4.00	2.00
35	8.00	5.00	4.00	0.00	2.00	2.00	4.00	2.00
40	9.00	5.00	4.00	0.00	2.00	2.00	5.00	2.00
45	10.00	6.00	5.00	0.00	3.00	3.00	5.80	3.00
50	10.00	7.00	5.00	0.00	3.00	3.00	6.00	3.00
55	11.00	8.00	5.00	0.00	4.00	4.00	7.00	3.00
60	12.00	9.00	6.00	0.00	4.00	5.00	7.00	4.00
65	13.00	10.00	6.00	0.00	5.00	6.00	8.00	4.00
70	14.00	11.00	7.00	0.00	6.00	7.00	9.00	5.00
75	16.00	12.00	7.00	0.00	7.00	8.00	10.00	6.00
80	16.20	14.00	8.00	1.00	8.00	9.00	10.00	7.00
85	18.00	15.00	8.00	2.00	9.00	10.00	11.40	8.00
90	20.00	18.00	9.00	3.00	11.00	12.00	13.00	10.00
95	23.00	21.00	10.00	5.00	13.00	14.00	16.00	13.00
99	27.00	26.00	12.00	11.00	19.00	18.00	20.00	16.00

Cisgender lesbian women (*N *= 573)								
	Body dissatisfaction	Binge eating	Cognitive restraint	Purging	Restricting	Excessive exercise	Negative attitudes toward obesity	Muscle building
*M* (*SD*)	13.61 (7.39)	8.82 (6.34)	4.82 (2.97)	0.77 (2.03)	4.79 (4.62)	3.9 (4.31)	5.08 (4.36)	2.22 (2.53)
Range	0–28	0–32	0–12	0–18	0–21	0–20	0–20	0–15
Rank								
5	2.00	0.60	0.00	0.00	0.00	0.00	0.00	0.00
10	4.00	1.00	1.00	0.00	0.00	0.00	0.00	0.00
15	6.00	2.00	2.00	0.00	0.00	0.00	0.00	0.00
20	7.00	3.00	2.00	0.00	0.00	0.00	1.00	0.00
25	8.00	4.00	3.00	0.00	1.00	0.00	1.00	0.00
30	9.00	4.60	3.00	0.00	1.00	0.60	2.00	1.00
35	10.00	5.00	3.00	0.00	2.00	1.00	3.00	1.00
40	11.00	6.00	4.00	0.00	2.00	2.00	3.00	1.00
45	12.00	7.00	4.00	0.00	3.00	2.00	4.00	2.00
50	13.00	8.00	5.00	0.00	3.00	3.00	4.00	2.00
55	14.00	9.00	5.00	0.00	4.00	3.00	5.00	2.00
60	15.00	10.00	5.00	0.00	5.00	4.00	6.00	2.00
65	17.00	11.00	6.00	0.00	6.00	4.00	6.00	2.00
70	18.00	12.00	6.00	0.00	7.00	5.40	7.00	3.00
75	19.00	13.00	7.00	0.00	8.00	6.00	8.00	3.00
80	20.00	14.00	7.00	1.00	9.00	7.00	9.00	4.00
85	22.00	16.00	8.00	1.20	10.00	8.00	10.00	4.00
90	24.00	17.00	9.00	3.00	11.00	10.00	11.00	5.00
95	26.00	21.00	10.00	5.00	14.00	13.00	13.00	8.00
99	28.00	25.56	12.00	10.00	17.28	17.00	18.00	11.28

Cisgender bi + men (*N* = 116)								
	Body dissatisfaction	Binge eating	Cognitive restraint	Purging	Restricting	Excessive exercise	Negative attitudes toward obesity	Muscle building
*M* (*SD*)	9.78 (5.95)	7.77 (6.29)	4.92 (2.81)	0.66 (1.87)	3.85 (4.06)	4.89 (4.62)	5.45 (4.18)	3.6 (3.64)
Range	0–28	0–32	0–12	0–14	0–23	0–20	0–15	0–17
Rank								
5	1.75	0.00	0.00	0.00	0.00	0.00	0.00	0.00
10	3.00	0.00	1.00	0.00	0.00	0.00	0.00	0.00
15	3.00	2.00	1.00	0.00	0.00	1.00	1.00	0.00
20	4.00	3.00	2.00	0.00	0.00	1.00	2.00	1.00
25	6.00	4.00	3.00	0.00	1.00	1.00	2.00	1.00
30	6.00	4.00	3.50	0.00	1.00	2.00	2.50	1.00
35	7.00	5.00	4.00	0.00	1.25	2.00	3.00	2.00
40	8.00	5.00	4.00	0.00	2.00	2.00	3.00	2.00
45	8.00	5.00	5.00	0.00	2.75	3.00	4.00	2.00
50	9.00	6.50	5.00	0.00	3.00	3.50	5.00	2.00
55	10.00	7.00	6.00	0.00	3.00	4.00	5.00	3.00
60	11.00	8.00	6.00	0.00	4.00	5.00	6.00	3.00
65	12.00	9.00	6.00	0.00	4.00	6.00	7.00	4.00
70	12.50	10.50	6.50	0.00	5.00	6.00	8.00	4.00
75	13.25	11.00	7.00	0.00	5.00	8.00	9.00	5.00
80	14.00	12.00	7.00	1.00	6.00	9.00	9.00	6.00
85	15.75	13.00	8.00	1.75	7.75	9.75	10.00	8.00
90	17.00	16.50	8.00	2.00	10.00	11.50	11.00	9.00
95	20.25	18.25	9.00	4.00	12.00	14.00	12.25	11.00
99	24.85	28.55	10.85	7.00	14.85	18.55	15.00	15.70

Cisgender bi+ women (*N* = 448)								
	Body dissatisfaction	Binge eating	Cognitive restraint	Purging	Restricting	Excessive exercise	Negative attitudes toward obesity	Muscle building
*M* (*SD*)	15.17 (6.89)	10.22 (7.09)	4.27 (2.99)	0.94 (2.35)	5.46 (5.28)	3.49 (4.28)	4.63 (4.53)	2.29 (2.78)
Range	0–28	0–32	0–12	0–21	0–22	0–20	0–20	0–17
Rank								
5	4.00	1.00	0.00	0.00	0.00	0.00	0.00	0.00
10	6.00	2.00	0.00	0.00	0.00	0.00	0.00	0.00
15	7.00	3.00	1.00	0.00	0.00	0.00	0.00	0.00
20	9.00	3.00	1.00	0.00	1.00	0.00	0.00	0.00
25	10.00	5.00	2.00	0.00	1.00	0.00	1.00	0.00
30	11.00	5.00	2.00	0.00	2.00	0.00	1.00	0.00
35	12.00	6.00	3.00	0.00	2.00	0.00	2.00	1.00
40	13.00	7.00	3.00	0.00	3.00	1.00	2.80	1.00
45	14.00	8.00	4.00	0.00	4.00	1.00	3.00	1.00
50	15.00	9.00	4.00	0.00	4.00	2.00	4.00	2.00
55	16.00	10.00	5.00	0.00	5.00	2.00	4.00	2.00
60	17.00	11.00	5.00	0.00	5.00	3.00	5.00	2.00
65	18.00	13.00	6.00	0.00	6.00	4.00	5.00	2.00
70	20.00	14.00	6.00	0.00	7.00	4.00	6.00	3.00
75	20.00	15.00	6.00	1.00	8.00	6.00	7.00	3.00
80	22.00	16.60	7.00	1.00	10.00	7.00	8.00	4.00
85	23.00	18.00	7.00	2.00	11.00	8.00	9.00	4.00
90	24.00	20.00	8.00	3.00	13.00	10.00	11.00	6.00
95	26.00	22.00	10.00	5.00	17.00	12.00	13.65	8.00
99	28.00	29.53	12.00	10.53	20.53	18.53	18.00	12.53

**Table 3:** Comparisons of the Eating Pathology Symptoms Inventory (EPSI) scales among cisgender gay men, cisgender lesbian women, cisgender bi + men, and cisgender bi + women

	Groups				Kruskal–Wallis test		Dunn’s test (post hoc)
	Cisgender gay men (a)	Cisgender lesbian women (b)	Cisgender bi + men (c)	Cisgender bi + women (d)	χ^2^	*p*	
	Median (IQR)	Median (IQR)	Median (IQR)	Median (IQR)			
Body dissatisfaction	10 (10)	13 (11)	9 (7.25)	15 (10)	129.21	< 0.001	c = a < b < d
Binge eating	7 (9)	8 (9)	6.5 (7)	9 (10)	26.13	< 0.001	c < a < b < d
Cognitive restraint	5 (4)	5 (4)	5 (4)	4 (4)	24.27	< 0.001	d < c = a = b
Purging	0 (0)	0 (0)	0 (0)	0 (0.1)	4.93	0.180	c = a = b = d
Restricting	3 (6)	3 (7)	3 (4)	4 (7)	11.17	0.010	c = a = b < d
Excessive exercise	3 (7)	3 (6)	3.5 (7)	2 (6)	44.00	< 0.001	d < a = b < c
Negative attitudes toward obesity	6 (7)	4 (7)	5 (7)	4 (6)	76.45	< 0.001	d = b < c < a
Muscle building	3 (4)	2 (3)	2 (4)	2 (3)	204.93	< 0.001	d = b < c < a

## Discussion

This study provides the first comprehensive EPSI community norms for cisgender sexual minority adults using data from a longitudinal national cohort study in the United States. In our analyses, cisgender bi + women showed the highest scores for three of the eight scales on the EPSI: Body Dissatisfaction, Binge Eating, and Restricting. We found cisgender bi + men to show the highest scores for Excessive Exercise, whereas cisgender gay men showed the highest scores on Muscle Building and Negative Attitudes Toward Obesity. Finally, we found that cisgender lesbian and bi + women scored higher on Body Dissatisfaction and lower on Negative Attitudes towards Obesity than cisgender gay and bi + men.

Our findings suggest that cisgender bi + individuals are at risk for greater disordered-eating symptoms compared to cisgender gay men and cisgender lesbian women. Previous literature has found that cisgender bi + individuals are at increased risk for a variety of mental-health conditions, including anxiety and depression, in comparison to their (presumably cisgender) heterosexual, gay, and lesbian counterparts [25]. These disparities are hypothesized to stem from the unique minority stressors faced by the bi + community, which are rooted in monosexism—the assumption that all individuals are attracted to only one gender [26]. Due to negative attitudes and stereotypes, such as questioning their sexual identity or being perceived as promiscuous or unfaithful, bi + individuals may feel pressured to conceal their identity or conform to the norms reinforced by monosexism [27]. They frequently report feeling less connected to the LGBTQIA + community, a dynamic that, combined with the aforementioned stressors, leads to poorer mental-health outcomes, including heightened anxiety and depressive symptoms [28]. This may contribute to maladaptive emotion regulation strategies, leading to higher rates of disordered-eating behaviors [29] among bi + individuals. In fact, one study found that anti-bisexual minority stress is uniquely linked to increased emotional eating and negatively associated with body esteem [30]. Interestingly, the same study revealed that affirmation and centrality of bisexual identity, along with LGBTQIA + community connectedness, act as protective factors for body esteem, emphasizing the crucial role of identity affirmation in intervention strategies.

While much of the research to date has examined the experiences of bi + individuals collectively, emerging evidence suggests that cisgender bi + men and women face unique challenges that may distinctly impact their eating-related concerns and behaviors. A substantial body of research has focused on the minority stress experiences of cisgender bi + women, particularly in relation to body image. Scholars have posited that bi + women may be at especially high risk for poor body image and disordered eating behaviors due to societal perceptions of bisexual women as hypersexual or sexually promiscuous [28, 31–33]. Bisexual individuals (presumed cisgender) have attributed these stereotypes not only to rejection by sexual partners, but also to increased targeting and sexual abuse [31]. In fact, studies have shown that bisexual women face significantly higher rates of sexual victimization compared to their heterosexual and lesbian counterparts [31, 34, 35]. Recent research has demonstrated that sexual orientation-based discrimination and objectification significantly impact (presumed cisgender) bisexual women’s body image and feelings of body shame [36, 37], reinforcing our finding that cisgender bi + women are at especially high risk for body dissatisfaction.

Our findings that cisgender bi + men showed the highest scores for Excessive Exercise and that cisgender gay men showed the highest scores for Muscle Building and Negative Attitudes toward Obesity appear to be consistent with prior literature. The limited research available on bi + men indicates a significant association between increased exercise frequency and disordered-eating symptoms, with bi + men being more likely than any other sexual minority subgroups to use exercise as a method of weight control [38]. As discussed previously, these findings may be attributed to the minority stress and biphobia experienced by bi + people, for whom disordered-eating behaviors may function as maladaptive coping mechanisms. Specifically, a desire for muscularity may contribute to the increased exercise frequency observed among bi + men [38]. With regard to the literature on (presumably cisgender) gay men, studies have repeatedly shown elevated body-image concerns and eating pathology among gay men relative to heterosexual men [39]. The drive for muscularity—coupled with the idealization of a lean, muscular body type—have been identified as contributing factors to the high prevalence of muscle-enhancing behaviors and dissatisfaction with muscularity among presumed cisgender gay men [40]. Intraminority stress theory, which posits that comparisons within the sexual minority male community (from peers and potential significant others) creates status-driven competitive pressures, has been suggested as a major factor contributing to the increased muscle-building behaviors observed among sexual minority men, specifically (presumed cisgender) gay men [41].

With regard to cisgender lesbian women, the literature on disordered eating appears mixed. Some findings suggest that presumed cisgender lesbian women experience lower rates of body dissatisfaction compared to heterosexual women [42, 43], whereas other studies have found no differences between rates of disordered-eating symptoms between those groups [17, 44–48]. Although our study did not compare cisgender lesbian and bi + women to their heterosexual peers, we found that cisgender lesbian and bi + women scored higher on Body Dissatisfaction and lower on Negative Attitudes Toward Obesity in comparison to cisgender gay and bi + men. Although body dissatisfaction and negative attitudes toward obesity both often stem from internalized societal beauty standards and weight stigma, the divergence in our results may be explained by different sources of body dissatisfaction in the context of cultural and community norms. For cisgender lesbian and bi + women, body dissatisfaction may be less about conforming to thinness ideals and more about feelings of inadequacy stemming from minority stress and stereotypes about their identities. In addition, although cisgender gay and bi + men may be more likely to internalize cultural norms that equate thinness with attractiveness, cisgender lesbian and bi + women may reject such norms as a form of resistance against heteronormative and patriarchal beauty standards [49], which could contribute to lower negative attitudes about obesity.

### Strengths and limitations

Notable strengths of this study include the provision of separate community norms for multiple distinct groups of cisgender sexual minority adults, as well as the focus on a newer multidimensional measure of eating pathology for which the assessment and psychometric literature are still limited. However, this study also has several limitations that should be acknowledged. Due to the community-based nature of the sample, scores across all EPSI scales were relatively low. Scores on the Purging scale were particularly low across all subgroups, which is consistent with the nonclinical nature of the sample. This may have constrained the study’s ability to detect statistically significant differences across certain EPSI scales. Although sexual orientation was not a specific focus of measure development of the EPSI, the overall purpose was to create a measure that was more inclusive across various realms of diversity than previous measures. Furthermore, the sample was predominantly composed of White and more highly educated individuals, which may limit the generalizability of our findings to more racially, ethnically, or socioeconomically diverse populations. Additionally, while the study offered inclusive response options for sexual orientation, participants who identified as queer, asexual/demisexual/gray-ace, or another sexual orientation were excluded from the current analysis due to conceptual differences, which may have limited the scope of our findings. The survey did not contain explicit attention checks; however, we believe the structured enrollment process, longitudinal participant engagement, and authentication protocols provide assurances regarding data quality and integrity. Future studies should focus on the queer, asexual/demisexual/gray-ace, and other underrepresented groups as they may be also at elevated risk for eating disorder symptoms.

## Conclusion

This study is the first to publish norms for the EPSI among cisgender sexual minority subgroups, including gay men, lesbian women, bi + men, and bi + women. These norms represent valuable benchmarks for interpreting EPSI scores among cisgender sexual minority individuals. Our results offer helpful data for clinicians, informing more targeted screening for eating pathology in vulnerable groups. Future research should examine differences in EPSI scores between cisgender sexual minority groups and heterosexual peers to further understand the factors contributing to disordered-eating symptoms across diverse sexual orientation groups and inform interventions to address these disparities. Future research should investigate the mechanisms driving higher rates of eating disorder symptomatology among bi + individuals and expand the demographic diversity of participants. Finally, additional studies are needed to examine how the prevalence and severity of disordered eating behaviors vary by sociodemographic factors, including race/ethnicity, age, and socioeconomic status, within sexual minority communities.

## What is already known on this subject?

Cisgender sexual minority adults are at higher risk for eating disorders and disordered eating behaviors compared to heterosexual individuals, driven by factors such as minority stress and societal stigma. Within sexual minority groups, the prevalence of specific eating disorder symptoms varies, with gay men and bisexual women being particularly vulnerable to conditions like anorexia nervosa and bulimia nervosa. Existing studies have validated certain eating disorder measures, like the Eating Disorder Examination-Questionnaire, in cisgender sexual minority populations. Despite advancements, there are limited data on eating pathology patterns stratified by specific cisgender sexual minority subgroups.

## What this study adds?

This study provides community norms for the EPSI among cisgender gay men, lesbian women, bi + men, and bi + women. Distinct patterns of eating pathology were identified; cisgender bi + women exhibited the highest levels of body dissatisfaction, binge eating, and restricting compared to other subgroups. Cisgender gay men demonstrated the highest scores for muscle building and negative attitudes toward obesity, while bi + men showed the highest levels of excessive exercise.

## Data Availability

Data from The PRIDE Study may be accessed through an Ancillary Study application (details at pridestudy.org/collaborate).
